# Supplementary material for: A Coarse-Grained Methodology Identifies Intrinsic Mechanisms That Dissociate Interacting Protein Pairs
Source: Front Mol Biosci. 2020 Aug 25;7:210. doi: 10.3389/fmolb.2020.00210 (PMC7477071; doi:10.3389/fmolb.2020.00210)
Supplement: Supplementary file 1 [file Table_1.pdf]

A Coarse-Grained Methodology Identifies Intrinsic Mechanisms That Dissociate Interacting Protein Pairs

Haleh Abdizadeh[1], Farzaneh Jalalypour[2], Ali Rana Atilgan[2] and Canan Atilgan[2*]

1 Groningen Biomolecular Sciences and Biotechnology Institute, University of Groningen, Groningen, Netherlands, 2 Faculty of Engineering and Natural Sciences, Sabanci University, Tuzla, Turkey

Table S1. Depths of residues singled out by PRS in the complex and unbound structures.

Type	Complex	PRS residue depth (Bound/Unbound) (Å)									
I	**1CLV(AI)**	**W57:A(10.5/ 6.6)**	N138:A(4.7/ 4.2)	**V151:A(8.1/ 3.8)**	**G152:A(5.5/ 3.6)**	G292:A(3.9/ 4.7)					
	**2OZA(AB)**	**L70:A(3.9/ 5.9)**	G71:A(3.4/ 3.3)	**G171:A(7.6/ 8.0)**	**Q175:A(5.0/ 5.0)**	Y176:A(3.9/ 4.3)					
	**1AVX(AB)**	**L520:B(5.7/ 5.5)**	K552:B(4.9/ 4.9)	S579:B(4.0/ 3.7)	F580:B(3.8/ 3.5)	A581:B(3.5/ 4.1)	D598:B(3.6/ 3.4)	K611:B(3.1/ 3.5)			
	**2A9K(AB)**	Y66:B(4.6/ 4.4)	G67:B(3.1/ 3.1)	L68:B(4.8/ 4.9)	S69:B(3.5/ 3.6)	**D112:B(8.1/ 4.2)**	S181:B(4.0/ 3.6)	F209:B(4.1/ 3.9)	A210:B(3.9/ 4.8)	G211:B(3.5/ 3.3)	
	**1AY7(AB)**	E8:B(4.8/ 4.2)	**L41:B(6.6/ 6.1)**	T42:B(4.0/ 3.9)	G43:B(3.8/ 3.3)	W44:B(3.7/ 4.0)	**E57:B(4.1/ 5.3)**	**Q58:B(4.6/ 5.6)**			
	**4CPA(AI)**	S134:A(3.7/ 3.7)	**K177:A(5.2/ 5.1)**	**S199:A(6.1/ 4.4)**	I274:A(4.8/ 4.4)						
	**1DFJ(EI)**	**L22:I(6.8/ 7.8)**	A46:I(4.7/ 4.6)	**L47:I(7.7/ 7.4)**	R48:I(4.9/ 4.7)	A49:I(3.9/ 3.6)	**G186:I(6.4/ 7.1)**	D213:I(4.2/ 4.2)	P450:I(4.2/ 3.9)	G451:I(3.3/ 3.4)	
	**1JK9(AB)**	C27:B(3.8/ 3.9)	**P54:B(6.5/ 6.6)**	S55:B(4.5/ 4.4)	D67:B(4.4/ 4.0)	**A68:B(5.6/ 5.7)**	I69:B(4.3/ 5.4)				
	**2ABZ(AC)**	**Q16:C(6.9/ 5.6)**	**V17:C(8.6/ 7.1)**	**C18:C(6.6/ 5.8)**	E31:C(4.8/ 3.5)						
	**1EWY(AC)**	**I62:A(8.9/ 9.7)**	V67:A(4.2/ 4.5)	D68:A(4.3/ 4.3)	K69:A(3.9/ 3.5)	**T164:A(11.3/ 11.9)**	**F183:A(5.8/ 6.9)**				
	**1PXV(AC)**	**V10:C(5.2/ 5.1)**	Y11:C(4.1/ 3.6)	H44:C(4.4/ 4.6)	**H54:C(4.7/ 5.3)**						
II	**1FFW(AB)**	G52: A (3. 9/ 3.7)	**V54:A(10.2/10.0)**	**D57:A(9.0/7.6)**	N59:A(4.5/4.4)	K190:B(4.2/4.4)	G191:B(3.3/3.5)	**L195:B(5.7/4.5)**	**A197:B(7.1/ 7.4)**		
	**1OFU(AX)**	**I207:A(6.8/4.4)**	**D210:A(4.0/6.7)**	**L271:A(7.0/5.3)**	S272:A(4.6/4.0)	**L87:X(3.9/5.5)**	T88:X(4.5/4.0)	**H89:X(6.8/4.2)**	**R93:X(6.2/7.1)**		
	**3D5S(AC)**	**R10:A(6.5/4.4)**	**L11:A(9.0/6.1)**	**K12:A(6.7/4.4)**	**H13:A(7.3/3.5)**	**L14:A(8.1/5.4)**	**I15:A(6.6/4.9)**	**V16:A(6.7/4.1)**	**T17:A(5.8/4.1)**	N67:C(4.7/4.7)	K70:C(3.6/4.2)	Q71:C(3.6/3.9)
	**1CGI(EI)**	A179:E(4.5/4.1)	**G197:E(14.6/9.7)**	T30:I(4.3/4.4)	**Y31:I(5.6/4.7)**	**P32:I(7.2/4.3)**					
	**1FLE(EI)**	L123:E(4.3/4.4)	**A208:E(5.2/5.6)**	**V209:E(7.2/8.0)**	T11:I(3.3/4.0)	K12:I(4.3/3.5)	P13:I(3.6/3.8)	L33:I(4.4/4.4)	K34:I(3.9/4.3)		
III	**1PHV(AB)**	**R276:A(7.5/ 7.6)**	**I277:A(9.1/ 9.1)**	E294:A(4.1/ 4.2)	A295:A(3.9/ 3.7)	S296:A(4.3/ 3.7)	G297:A(4.1/ 3.8)	G147:B(4.8/ 4.1)	P148:B(3.5/ 3.7)	D149:B(3.8/ 4.0)	T150:B(3.2/ 4.4)
	**1JIW(PI)**	N191:P(4.6/ 3.6)	**A192:P(5.4/ 3.5)**	G193:P(3.4/ 3.5)	E21:I(3.8/ 4.0)	A22:I(3.6/ 3.7)					
	**1US7(AB)**	A97:A(4.3/ 4.1)	A244:B(4.1/ 3.4)								
	**1D6R(AI)**	**G193:A(6.6/ 5.8)**	H33:I(4.4/ 4.1)	S34:I(3.6/ 3.5)							
IV	**2OUL(AB)**	**I68:A(7.3/7.0)**	S113:A(4.4/4.6)	**V114:A(5.5/5.4)**	D148:A(3.9/4.7)	**F219:A(7.8/7.7)**	**Y37:B(7.5/7.6)**	G41:B(4.4/4.4)			
	**1R6Q(AC)**	**E7:A(7.0/4.9)**	E73:A(3.9/3.6)	**S118:A(6.9/3.8)**	**Y122:A(6.2/4.2)**	D45:C(4.1/ 4.2)	K49:C(4.5/3.8)	**L61:C(4.7/ 5.2)**			
	**2OOR(AC)**	**A166:A(5.6/ 3.6)**	L214:A(4.2/ 3.9)	T220:A(4.3/ 4.2)	N103:C(4.0/ 3.8)	P105:C(3.9/ 4.1)	M167:C(4.0/3.5)				
	**1GL1(AI)**	**C58:A(5.3/5.4)**	S76:A(3.6/ 3.4)	S77:A(3.7/3.9)	K13:I(3.8/3.6)	C14:I(3.9/3.8)					
	**1BVN(PT)**	Q5:P(4.1/ 4.3)	**T6:P(4.9/ 5.0)**	Q7:P(3.8/ 4.3)	S8:P(3.5/ 3.8)	**R10:P(4.9/ 5.2)**	V804:T(3.4/ 3.7)	C811:T(3.7/ 3.8)	**A823:T(8.5/ 7.3)**		
